# Sensitive Displacement Sensor Based on a Flexible Grating Random Laser

**DOI:** 10.3390/nano15211605

**Published:** 2025-10-22

**Authors:** Guang Dai, Yan Liu, Zhenzhen Shang, Yangjun Yan, Hui Peng, Heng Zhang

**Affiliations:** 1 Tianjin Key Laboratory of Quantum Optics and Intelligent Photonics, School of Science, Tianjin University of Technology, Tianjin 300384, China; daigersky@163.com; 2 Tianjin Key Laboratory of Autonomous Intelligence Technology and Systems, School of Computer Science and Technology, Tiangong University, Tianjin 300387, China; 3 School of Science, Qiongtai Normal University, Haikou 571127, China; 4 Key Laboratory of High Performance Scientific Computation, School of Science, Xihua University, Chengdu 610039, China; 5 School of Resources, Environment and Materials, Guangxi University, Nanning 530004, China; penghuimaterial@163.com

**Keywords:** random laser, PDMS, flexible, sensor

## Abstract

This study proposes and demonstrates a highly sensitive displacement sensor based on a flexible random laser. The sensor utilizes a polydimethylsiloxane (PDMS) film where a self-assembled surface grating structure is formed via oxygen plasma surface treatment combined with bending prestress. This structure acts as a photon-trapping microcavity and multiple scattering feedback center, integrated with embedded laser dye PM597 as the gain medium to form a flexible grating random laser. Experiments show that the device generates random lasing emission under 532 nm pumping (threshold ~21 mJ/cm^2^) with a linewidth of ~0.25 nm and a degree of polarization of ~0.82. Applying micro-displacement alters the PDMS film curvature, subsequently changing the grating morphology (height, angle). This modifies photon trapping efficiency and geometric deflection loss within the equivalent resonator cavity, leading to significant modulation of the random laser output intensity. A linear correspondence between displacement and lasing intensity was established (R^2^ ≈ 0.91), successfully demonstrating displacement sensing functionality. This scheme not only provides a low-cost method for fabricating flexible grating random lasers but also leverages the extreme sensitivity of random lasing modes to local disordered structural changes, paving the way for novel high-sensitivity mechanical sensors and on-chip integrated photonic devices.

## 1. Introduction

Random lasers (RLs) leverage disordered gain media with multiple scattering centers to amplify light through closed-loop feedback pathways, bypassing the need for conventional optical cavities [1,2,3,4,5]. This unique mechanism grants RLs advantages in fabrication simplicity, spectral flexibility, and robustness to structural imperfections. Recently, the phenomenon of random lasing generated by electric pumping has been realized in the laboratory [6], which offers new ideas for the development of novel weakly coherent light sources due to their application prospects in speckle-free imaging [7]. However, the inherent stochasticity of photon transport paths impedes precise control over lasing parameters (e.g., wavelength, intensity) [8,9,10,11] and complicates establishing deterministic correlations between external stimuli and spectral outputs—a critical barrier for sensor development.

Traditional approaches to RL modulation, such as polymer-stretch-aligned liquid crystal scatterers [12,13], bio-photonic crystal [14,15], face material limitations. Polyvinylidene fluoride (PVDF), while mechanically robust, suffers from poor elastic recovery, restricting device reusability [16]. Polydimethylsiloxane (PDMS) emerges as a superior alternative, offering high transparency (>95% in the visible range), exceptional stretchability (>100% strain), near-perfect shape recovery, and facile micro/nanofabrication compatibility. These attributes underpin its dominance in flexible photonics, including polymer gratings [17,18], microfluidics, and optoelectronic devices.

The integration of PDMS with microfluidics has catalyzed optofluidic technologies, enabling dynamic reconfiguration of optical components—a feat unattainable in rigid solid-state systems. While conventional lasers often rely on complex external cavities or distributed gratings for feedback, their fabrication demands stringent alignment precision. RLs circumvent this by utilizing intrinsic disorder for coherent emission, though achieving reproducible and tunable disorder remains challenging. Recent strategies employ engineered scattering substrates (e.g., crumpled graphene oxide, planar fluorine-doped tin oxide (FTO) substrates, and patterned sapphire substrates (PSSs)) [19,20], yet flexible, real-time tunable platforms are scarce.

To overcome these challenges, we propose a displacement sensing paradigm based on a flexible random laser featuring a dynamically tunable PDMS surface grating. Our approach exploits oxygen plasma surface modification under controlled bending pre-stress to achieve spontaneous self-assembly of corrugated gratings (period ~1.1 μm, duty ratio ~0.7). These structures serve as reconfigurable multi-feedback centers, while dye molecules (PM597) embedded within the PDMS matrix act as the gain medium. Crucially, micro-displacement-induced curvature changes modulate grating morphology (height, ridge angle), thereby altering photon trapping efficiency within the equivalent micro-cavity via geometric deflection loss. This mechanism enables direct modulation of RL output intensity by mechanical input. The hydrophobic PDMS encapsulation further ensures environmental stability. This work establishes an alignment-free route to flexible grating RLs and pioneers their application in displacement sensing by harnessing the intrinsic sensitivity of random lasing modes to local disorder perturbations.

## 2. Materials and Methods

This article investigates feedback-enhanced stochastic laser sensing via low-cost PDMS surface grating fabrication. The high flexibility of PDMS enables responsive modulation of grating morphology under external stress, distortion, or displacement, thereby tuning random lasing properties. PDMS gratings are fabricated through oxygen plasma surface treatment, offering a cost-effective alternative to conventional lithography techniques (e.g., ion beam lithography, laser direct writing, or 3D printing [21]).

**Figure 1:**
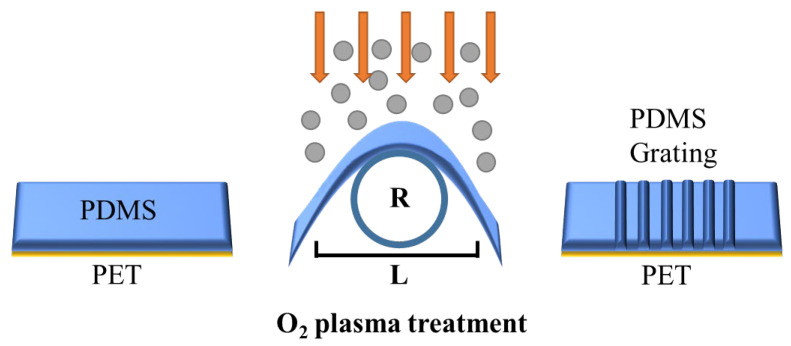
Schematic diagram of sample preparation.

The production of PDMS encoders is divided into seven steps. (1) Mix the PDMS polymer with the curing agent with a mass ratio of 10:1, stir for 30 min until fully mixed, and then let it stand in the air for 1 h until the internal bubbles are removed. Otherwise, the internal bubbles will seriously affect the tensile properties of the film after film formation, and the uneven internal refractive index will also cause strong scattering and diffraction losses, which will affect the optical properties of the device. (2) The PDMS solution was slowly added dropwise on a long PET substrate with a length of 3 cm, a width of 1 cm and a thickness of 0.5 mm, evenly spun-coated at a speed of 1000 rpm, and then allowed to stand at room temperature for 20 min to remove air bubbles. (3) The substrate is placed on a hot plate and heated and cured at 70 °C under atmospheric pressure to obtain PDMS&PET double-layer film. (4) Give the double-layer film a certain bending prestress, use dovetail clamps to clamp both ends of the film, one side of the PDMS film protrudes upward, and adjust the depth inserted into the dovetail clamp to adjust the radius of curvature of the film bending. (5) The sample was placed in an oxygen plasma cleaning machine and discharged for surface oxidation for 10 min. After exposure to O_2_ plasma, the silane (Si-CH_3_) group on the surface of PDMS is converted to a silanol (Si-OH) group, and the surface becomes hydrophilic. The resulting silanol group may condense with adjacent silanol groups to form a brittle layer in which silicon may be bonded to three or four oxygen atoms (SiO_x_) [22,23]. (6) Slowly release the pre-stress to make the film tend to be flat. Due to the mismatch of the stiffness of the upper and lower surfaces of the PDMS film, it will self-assemble on the surface of the hard layer to form a corrugated grating structure. (7) The two ends of the double-layer membrane are attached to the substrate of the glass slide of the same size to be fixed, and a layer of ethanol solution of dye PM597 is spun on the surface. The device is finished. The processing and preparation process is shown in Figure 1.

In addition, a small amount of toluene solution of PM597 can be directly added to the PDMS solution in step (1), and the subsequent steps are the same. It will obtain a pleated grating device embedded with a luminescent gain medium.

The size of the grating structure is affected by the radius of curvature under prestress, the time of oxygen plasma treatment, the oxygen flux, the radio frequency power, and other factors. The amplitude ***A*** and period d of the obtained pleated structure can be obtained based on the linear buckling analysis of the pleated structure model of the self-assembly of the rigid film and the flexible substrate [24].

(1) $$\begin{array}{l} d=2\pi h_{f}{\left[\frac{E_{f}(1-\nu_{s}^{2})}{3E_{s}(1-\nu_{f}^{2})}\right]}^{\frac{1}{3}}\\ A=h_{f}\sqrt{\frac{\varepsilon_{\mathrm{pre}}}{\varepsilon_{c}}-1} \end{array}$$

where ***ν*** is the Poisson’s ratio (0.5 for PDMS), ***h*** is the thickness of the film, and ***E*** is the Young’s modulus. The subscripts f and s denote the rigid film on the surface and the flexible substrate on the bottom, respectively.

where ***h_f_*** is thickness of the stiff surface layer, ***E_f_*** and ***E_s_*** are Young’s modulus of the stiff layer and PDMS substrate, ***ν_f_*** and ***ν_s_*** are their Poisson’s ratios. ***ε_pre_*** is prestress, ***ε_c_*** is critical stress. Using our experimental parameters (***h_f_***∼150 nm, ***E_f_***∼10 GPa, ***E_s_***∼1.5 MPa, ***h_f_***∼150 nm, **E_f_**∼10 GPa, ***E_s_***∼1.5 MPa), Equation (1) predicts a period of ∼1.04 μm, which agrees well with our measured value∼1.1 μm (see Section 3).

## 3. Results and Discussion

**Figure 2:**
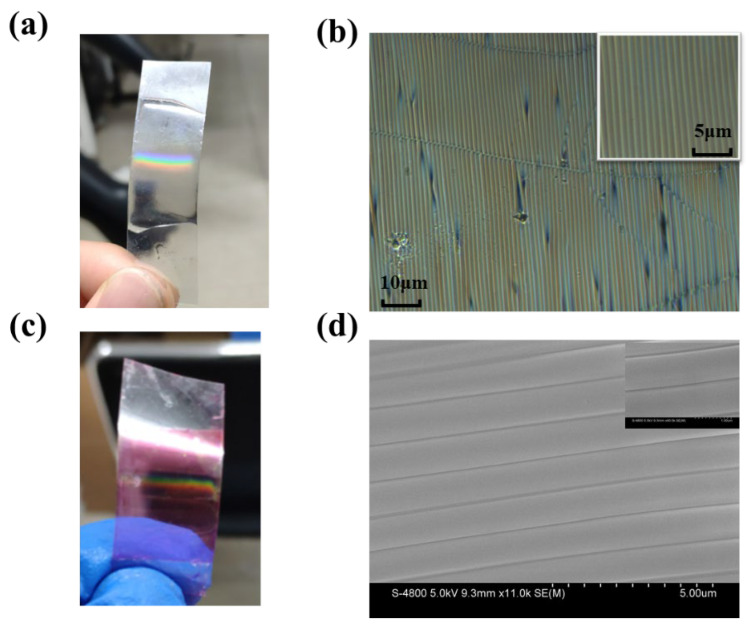
Actual grating and optical microscope image. (**a**) Physical drawing of pure polymer grating; (**b**) Light microscope photographs, with a partial enlarged illustration in the upper right corner; (**c**) Physical diagram of the dye polymer grating; (**d**) SEM test chart with 5 μm scale, local enlarged image with 2 μm scale in the upper right inset.

In this experiment, the curvature of the film was 0.5 cm, the internal air pressure of the plasma box was about 20 Pa, the RF power was 300 W, and the oxygen plasma treatment time was 600 s. Figure 2a is the actual image of the PDMS film treated under a fluorescent lamp, and we can observe the obvious color-banded stripes, which indicate that a parallel grating structure has formed on the surface of the PDMS.

Figure 2b shows a 60× magnification optical microscope photograph showing that a relatively uniform strip grating structure has been formed on the surface of the PDMS. The grating period is about 1.1 μm, and the duty ratio is about 0.75. Figure 2c shows that a dye-doped PDMS grating prepared by the same method shows similar diffraction fringes can be observed under light, demonstrating that a similar grating structure has been formed on the surface. Local voiding defects are observed, which primarily result from the rapid stress release during pre-strain recovery or mechanical distortion that compromises the structural integrity of the grating ridges. Representative scanning electron microscopy (SEM) characterization, presented in Figure 2d, confirms the formation of the surface grating, revealing a local period of approximately 1 μm and a duty ratio (defined as the ridge width-to-period ratio) of ~0.8. These structural parameters are in good agreement with the values derived from optical microscopy analysis.

**Figure 3:**
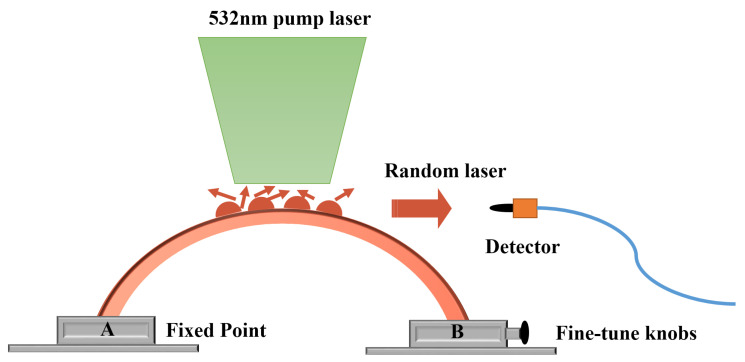
Schematic diagram of the structure of the flexible grating random laser sensing system.

In order to verify the feasibility of this scheme, we built a micro-displacement sensing system as shown in Figure 3. Fix both ends of the PET substrate on a precision translation stage with the PDMS grating side facing up. By adjusting the knob of the translation stage, the distance between the ends of the bent film can be precisely adjusted. The sample was pumped using a 532 nm nanosecond laser (~8 ns, 10 Hz). The incident laser beam is focused on the central area of the fabricated sample in a strip shape of 1cm length and 20 μm width by a cylindrical lens along the normal line of the sample. The fiber spectrometer detected a random laser signal in a direction nearly parallel to the grating array. As shown in Figure 3, it is assumed that points A and B are fixed points.

In the designed PDMS flexible grating random laser system, PM597 acts as a laser gain medium evenly distributed inside the thin film and is protected by a superhydrophobic polymer, and the device has excellent environmental stability. The flexible grating waveguide structure acts as a multi-scattering substrate. The large amount of light scattered between the wrinkled waveguide ridge and the trough can overcome the energy loss due to reflection, resulting in a coherent burst of photons that leads to laser action. Different from the stochastic laser device in which the liquid crystal is the scattering center in the previous paper, the flexible grating random laser prepared by this scheme will not be disturbed by external temperature, electromagnetic field, or other factors.

**Figure 4:**
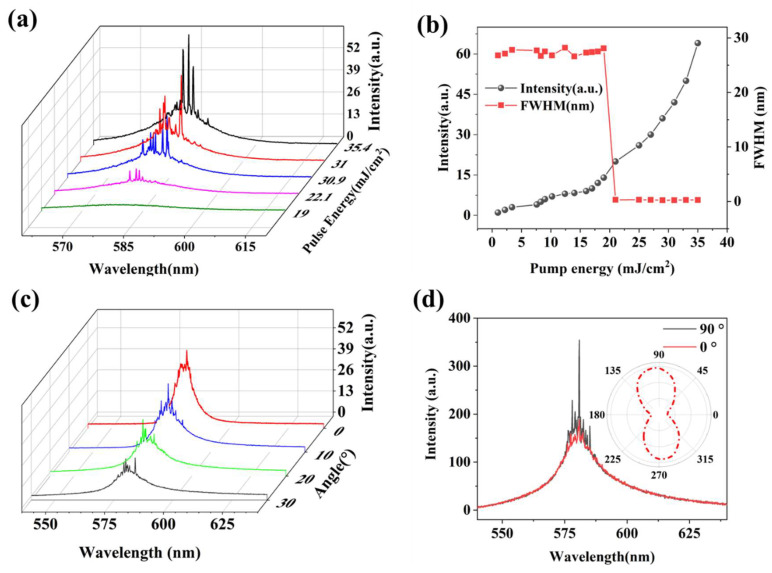
Random laser testing. (**a**) Random laser spectra at different pump powers; (**b**) Spectral intensity and full width at half maximum as a function of pump energy density; (**c**) Random laser spectra at different detection angles; (**d**) Degree of polarization testing.

First, the film is kept in a near-parallel state with obvious diffraction fringes visible to the naked eye, and then the pump light source is turned on to excite the sample, and the spectrum is recorded on a computer. Figure 4a shows the emission spectra at different pump energy densities, where emission peaks cover from ~550 nm to ~600 nm. Figure 4b shows the integral intensity of the spectrum and the line width (FWHM) as a function of the pump energy density. It can be seen that at a lower pump energy density, the device exhibits a wide emission peak similar to that of photoluminescence (PL) emission spectrum, and when the pump energy density is increased, multiple obvious spikes with line widths less than 1 nm are superimposed on the PL emission spectrum, and the number of spikes, i.e., the number of random laser modes, continues to increase with the further improvement of pump energy density. When the pump energy density reaches about 21 mJ/cm^2^, the spectral integration intensity increases rapidly, and the half-peak width of the spectrum decreases rapidly from about 27 nm to about 0.25 nm, which is completely consistent with the random laser. In order to further confirm that the detected spectrum is random laser radiation and not the distributed feedback laser radiation generated by the Bragg grating, the pump power is kept at about 25 mJ/cm^2^, the fiber probe is pointed at the same position of the film, so that the direction of the fiber probe is at an angle of 0–30° with the horizontal line, and the results are collected at 10° intervals, as shown in Figure 4c, similar random laser spectra can be obtained from different detection angles, and the wavelength and number of spikes are different from each other. This just verifies the unique performance of the multi-angle output of the random laser, and the random laser in each output direction corresponds to a different lasing mode.

The polarization of laser exit is also an important hallmark of random lasers, which has been verified in earlier years [25,26]. The polarization direction of the pump light is parallel to the grating, keeping it unchanged, a polarizer is placed between the sample and the fiber probe, and the laser with the same polarization state as the transmission direction of the polarizer is allowed to pass through the angle of the rotating polarizer, and the polarization degree of the random laser is calculated by comparing the maximum and minimum values of the random laser intensity during the rotation process. In this experiment, the polarizer transmission direction is defined to be 0° when it is parallel to the grating alignment direction, and the test results are shown in Figure 4d. When the polarizer transmission direction is 90°, that is, when the polarization direction is perpendicular to the grating direction, the signal intensity of the random laser is the largest, and when the polarizer transmission direction is 0°, there is only a weak random laser signal. As we all know, the formula for calculating the degree of polarization P is

(2) $$P=\frac{I_{\max}-I_{\min}}{I_{\max}+I_{\min}}$$

where *I*_max_ is the maximum intensity detected, and *I*_min_ is the minimum intensity. According to the test results, the grating random laser has a high degree of polarization, about 0.82. Therefore, the random laser light source produced by this method is expected to be applied in many fields such as polarized light display, infrared medical treatment, polarized imaging, film and television, etc.

Highly ordered grating structure could support DFB-like emission; however, we believe that the emission behavior observed in our device is consistent with a random laser due to the following reasons:

Firstly, a classic DFB laser relies on a highly periodic structure to provide distributed feedback via Bragg scattering. This creates a photonic bandgap, and lasing occurs at the band edges in well-defined, extended modes. In our device, while the grating is periodic on a macroscopic scale, it possesses inherent imperfections, local variations in period, duty cycle, and ridge height (as visible in Figure 2b,d), and most importantly, it is dynamically deformable. These imperfections break the long-range order required for a pure DFB mechanism. Instead, they create a large number of localized cavities or “hot spots” along the grating. Each of these localized regions, defined by a specific local configuration of grating ridges, can trap light and act as a resonant cavity. The sharp, well-resolved peaks correspond to the lasing modes of these individual, high-Q random cavities.

Secondly, as shown in Figure 4c, similar random lasing spectra are obtained from different detection angles, with spike wavelengths and numbers varying significantly. This is inconsistent with conventional DFB lasers, which typically exhibit directional emission. The random lasing spikes observed in Figure 4a,c vary in both wavelength and number with changes in pump position and detection angle—a hallmark of random lasing.

Lastly, in the figure, we can observe that when bending causes changes in the grating structure, the wavelength of the laser spike does not exhibit continuous movement, but rather shows an alternating pattern of increase and decrease. In the case of a DFB laser, the number of spectral modes does not change, and the wavelength exhibits corresponding continuous shifts as the structure changes continuously.

After demonstrating the randomness of the observed laser lasing, we further explored the sensitivity of the local perturbations. The effect of disordered structure changes on random laser emission has not been experimentally studied, as it is often very difficult to accurately control disordered structures locally.

**Figure 5:**
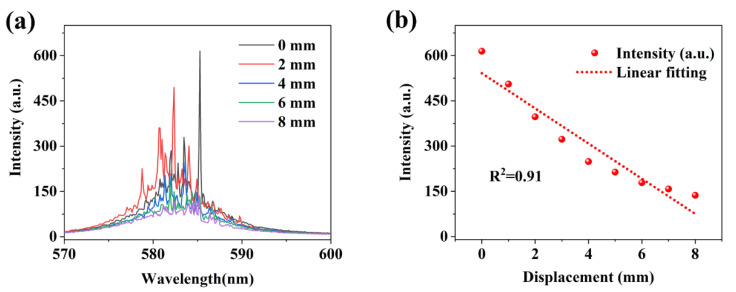
Random laser displacement sensing based on changes in intensity. (**a**) Relationship between the displacement and the random laser intensity; (**b**) Correspondence between the displacement of point B and the spectral intensity.

In order to verify the sensing performance of the grating feedback random laser, we tuned the displacement of point B and detected the spectral characteristics of the random laser in real time to establish the correspondence between the two. The pump light energy is maintained at 50 mJ/cm^2^, and the initial displacement of 0 corresponds to the initial AB point spacing of 2 cm and the micro-displacement stage at the bottom of point B gradually approaches point A, and the intensity change in the spectrum is recorded at the same time, and the results are shown in Figure 5.

Figure 5a shows the relationship between the displacement and the random laser intensity as the distance between the two points of AB is close, and it can be found that the random laser intensity shows a significant downward trend with the movement of point B. When the displacement near the bottom AB point reaches 8 mm, the sharp random laser radiation is almost impossible to observe. This has a lot to do with the feedback path of the random laser, and the stimulated emission amplification in this sample is due to the gain caused by multiple feedback loops back and forth on the grating end face. Photons located in the grating valley are reflected multiple times between the grating ridges, resulting in photon capture. When the distance between the AB points decreases, the curvature of the test points becomes smaller, the surface of the PDMS film tends to be flattened, the height of the grating decreases, the angle between the gratings becomes larger, and the feedback of photons is more likely to overflow outside the equivalent cavity, which greatly increases the geometric deflection loss of the equivalent resonator. Due to the presence of a feedback loop in the grating, the total energy loss due to multiple scattering should be overcome by optical gain. Feedback loops can be more easily implemented when the optical mean free path (which is approximately equal to the fold width) ≤ λ. Therefore, when point B is close to point A, the random laser intensity decreases, and conversely, when point B is far away from point A, the grating ridge is closer, the equivalent cavity length becomes shorter, the random feedback is enhanced, and the random laser intensity increases. Figure 5b shows the correspondence between the displacement of point B and the spectral intensity, which we linearly fitted with a linearity of R^2^ ≈ 0.91.

## 4. Conclusions

This paper prepares a flexible polymer grating through a simple oxygen plasma surface treatment method, studies the impact of random lasers caused by local changes in the surface grating due to micro-displacement, and designs a displacement sensor for flexible grating random lasers based on the influence of changes in the photon capture microcavity provided by the grating on the output intensity of random lasers. This scheme well demonstrates that the patterns of scattering random media are very sensitive to the position of the medium or to disordered local changes in local optical properties. Our experiments show that the combination of fluid and optical functions in optofluid random lasers is particularly suitable for exploring the sensing capabilities of random lasers. This greatly simplifies the design of on-chip integrated lasers, while providing new ideas for the application of active optofluid random laser sensing devices. Because intracavity scattering losses can lead to photon energy loss, thermal effects, and other factors that traditional laser devices strive to avoid, while random lasers require gain materials to not only have high gain but also exhibit high thermal stability and low self-absorption effects (large Stokes shift), dyes are more suitable as the gain medium for random lasers. From the results of the linear correspondence between output change and input change, random lasers have a sensitive response speed and the potential for mechanical sensing applications. However, due to the randomness of the scattering path, it is difficult to establish a linear correspondence between mechanical quantities and laser spectral parameters, indicating there is still room for research and optimization.

## Data Availability

The original contributions presented in this study are included in the article. Further enquiries should be directed to the corresponding author(s).
